# Torque Teno Virus (TTV) distribution in healthy Russian population

**DOI:** 10.1186/1743-422X-6-134

**Published:** 2009-09-07

**Authors:** Evgeny V Vasilyev, Dmitry Y Trofimov, Alexander G Tonevitsky, Valery V Ilinsky, Dmitriy O Korostin, Denis V Rebrikov

**Affiliations:** 1 DNA-Technology JSC, Kashirskoe shosse, 23-5-16, Moscow, 115478, Russia; 2 All-Russian Institute of Physical Culture and Athletics, Elizavetinsky lane, 10, Moscow, 105005, Russia; 3 Vavilov Institute of General Genetics, Gubkina st, 3, Moscow, 119991, Russia

## Abstract

**Background:**

Torque teno virus (TTV) is a circular, single-stranded DNA virus that chronically infects healthy individuals of all ages worldwide. There is a lot of data on the prevalence and genetic heterogeneity of TTV in healthy populations and in patients with various diseases now available. However, little is known about TTV load among healthy human population. In this study we analyzed TTV load in the group of 512 Russian elite athletes, who are supposed to be, by some standards, the healthiest part of the human population.

**Results:**

The prevalence rate of TTV among the Russian Olympic Reserve members was 94% (for test sensitivity about 1000 genome equivalents per 1 ml of blood). Quantities varied from 10^3 ^(which corresponded to detection limit) to 10^10 ^copies per 1 ml of blood, with median at 2.7 × 10^6 ^copies.

**Conclusion:**

About 94% of healthy individuals in Russian population have more than 1000 TTV genome copies per 1 ml of blood. This result exceeds the previously published data, and can be explained by either more sensitive PCR test system or by higher TTV distribution in Russian population or both. TTV viral load neither depends on gender, nor age.

## Background

Torque teno virus (TTV) was first discovered in 1997 in Japanese patients with non-A-G transfusion-acquired hepatitis [1]. TTV is a small, non-enveloped virus with a single-stranded, circular DNA genome of negative polarity, 3.4-3.9 Kb in length, containing two bigger (ORF1 and ORF2) and several smaller open reading frames [2]. TTV is currently classified to Circoviridae family [2]. Despite being a DNA virus, TTV demonstrates an extremely wide sequence divergence. At least 16 genotypes with evolutionary distance >0.30 has been described so far [3].

TTV is an ubiquitous virus revealed in more than 50% of the general human population throughout the world [4-6] and nearly 90% of pongid populations [7]. Co-infection of single individuals with TTV isolates belonging to one or several phylogenetic groups occurs frequently [8]. TTV was first characterized as a blood-born virus and thus referred to transfusion-transmitted (TT) group of viruses [1]. Recent studies suggested the existence of other ways of transmission including parenteral [3], sexual [9,10], mother-to-child [11,12] and others [13-15].

TTV has been suggested to be a causative agent of several diseases such as acute respiratory diseases [16], liver diseases [5,6], AIDS [17] and cancer [18], however, without any convincing support. One of current hypothesis suggests a key role of TTV in development of autoimmune reactions [19].

Despite 10 years of investigation, the TTV distribution in humans is still a subject of discussion. Possibly, this is because of the variability of TTV genotypes and the inability to design a single set of PCR primers, corresponding to the vast majority (if not all) viral genotypes [20]. Also little is known about distribution of TTV in healthy adults [21,22]. In this study we analyzed TTV viral load in blood of 512 Russian elite athletes who represents healthy Russian population.

## Results

**Figure 1:**
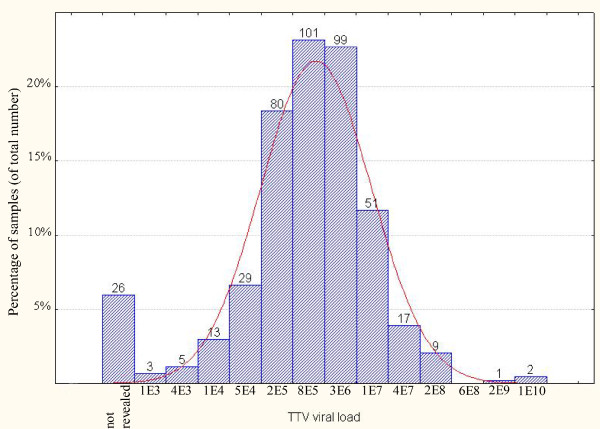
**Viral load of TTV among studied samples**. The histogram shows distribution of viral load among studied samples. Numbers above each bar represent number of samples.

Using qPCR we demonstrated that 485 out of 512 (94%) healthy individuals have TTV viral load of more than 1000 copies per 1 ml of blood, which corresponds to 94.1% of males and 93.9% of females studied. Considerable part of the athletes (39.9%) had viral load about 10^6 ^copies per 1 ml of blood (median 2.7 × 10^6^) with maximum about 10^10 ^viral genomes per 1 ml (see Fig. 1).

We failed to detect any correlation between the viral load and the age of tested individuals (R = 0.020; p > 0.05). Also no difference was detected in viral load for men and women (independent t-test: t-value = -1.943; p = 0.052).

## Discussion

Discovered frequency of TT virus presence in top rank Russian athletes exceeds the previously published data for healthy human populations (14-88%) [1-6,15,16,21]. The difference may be explained by wider distribution of TTV in Russians and/or more sensitive PCR test system, which detects more TTV variants. The latter idea is supported by the fact that the frequency of TTV in populations detected in the recent studies is higher than in the early studies, which probably can be explained by the application of more accurate methods for TTV detection. In fact, the TTV genome has estimated sequence divergence of more than 30% [15], and the minority of methods used so far were able to detect all the TTV variants. Expansion of the TTV sequencing data enabled us to design primers corresponding to the most constant sites in the TTV genome.

Our PCR test system has calculated sensitivity about 1000 viral genomes per ml of blood. Thus, all the samples with the less viral load were considered as negative. Our data may suggest that the real frequency of TTV presence in human population tends to be close to 100%.

## Conclusion

TTV viral load of more than 1000 copies per 1 ml of blood was detected for 94% of Russian elite athletes. Maximum viral load was about 10^10^, median was 2.7 × 10^6^. There were no significant differences between men and women. Also viral load did not seem to be dependent on age (in the range of this study). In general, the presence of TTV in healthy human population was higher than it has been previously described in literature. This fact can be explained by the higher presence of TTV among Russian population and/or by more sensitive PCR test system used. The absence of correlation between the age and the viral load supports the previously published data.

## Methods

### Samples and DNA purification

We analyzed the venous blood of 205 men and 232 women of Russian elite athletes aged between 12 and 36. All samples were stored less than three hours at +4°C before analysis. DNA was extracted from blood samples by standard phenol-chloroform extraction [23]. To prevent PCR contamination by blood samples or DNA samples, DNA isolation was performed in a separate DNA-extraction room (Zone 1). To prevent samples cross-contamination, all the procedures were carried out with sterile disposable tubes and aerosol-resistant tips in UV-equipped PCR-box.

### Primer and probe design and sequences

The primers and the probe for PCR were designed using the Oligo Primer Analysis software v6.31 (Molecular Biology Insights Inc., USA) and mfold v3.1 [24] based on alignment of different TTV genome sequences (GenBank, [25]). The set of primers used in this study amplify all known TTV variants (for primer and probe sequences see Table 1).

**Table 1:** Primers and probe sequences

**Name**	**Sequence**
TTV_s	5'-CCT GCA CTT CCG AAT GGC TGA GTT-3'

TTV_as	5'-CTT GAC TGC GGT GTG TAA ACT CAC-3'

TTV-tqm	5'-FAM-CCT CCG GCA CCC GCC CTC GGG ACG-BHQ1-3'

### qPCR

qPCR was used for detection and quantification of TTV load. For each PCR sample contained 1-μl-blood equivalent, we may estimate test sensitivity as 1000 viral copies per 1 ml of blood (with PCR sensitivity of 1 target per PCR tube).

PCR was carried out in reaction volumes of 35 μL with 80 mM Tris-HCl pH 8.6, 20 mM (NH_4_)_2_SO_4_, 3 mM MgCl_2_, 200 μM of each dNTP, 300 nM of each primer, 75 nM of probe and 2.0 U of Taq-polymerase. Real-Time PCR was performed using DT-96 Real-Time PCR Cycler (DNA-Technology, Russia) with the first denaturation step of 60 s at 94°C, followed by 50 cycles at 94°C for 10 s, 64°C for 20 s, 72°C for 10 s, with fluorescence reading at 64°C. The delta-TF method was used for curve normalization [26] and ΔΔCp for quantification. Human MTHFR gene was used for DNA amount standardization.

To prevent PCR contamination by previous reactions nor biological samples, all the reactions were combined using aerosol-resistant tips in UV-equipped PCR-box in a separate PCR-preparation room (Zone 2). Also, no electrophoresis (or other procedures with PCR-tube opening) was done with TTV PCR primers in this building. All the negative controls and surface washings were negative.

### Data analysis

PCR data was analyzed using DT-96 Real-Time PCR Cycler Software v.7.2 (DNA-Technology, Russia). Statistica 8.0 (StatSoft, USA) was used for statistical analysis.

## Competing interests

The authors declare that they have no competing interests.

## Authors' contributions

EVV performed all the experimental procedures and interpreted the data. DYT designed the primers. AGT arranged the sample collections and drafted the manuscript. VVI interpreted the data and drafted the manuscript. DOK interpreted the data. DVR designed the study and wrote the manuscript. All authors read and approved the final version of manuscript.
